# Using an Internet-Based Hospital to Address Maldistribution of Health Care Resources in Rural Areas of Guangdong Province, China: Retrospective and Descriptive Study

**DOI:** 10.2196/medinform.9495

**Published:** 2018-12-21

**Authors:** Cui He, Qiru Zhou, Wei Chen, Junzhang Tian, Lihua Zhou, Hong Peng, Shuo Luan, Shengfei Wang

**Affiliations:** 1 Department of Rehabilitation Guangdong Second Provincial General Hospital Guangzhou China; 2 Department of Guangdong Online Hospital Guangdong Second Provincial General Hospital Guangzhou China; 3 Outpatient Department Guangdong Second Provincial General Hospital Guangzhou China; 4 Guangdong Second Provincial General Hospital Guangzhou China; 5 Nursing Department Guangdong Second Provincial General Hospital Guangzhou China; 6 Department of Otorhinolaryngology, Head and Neck Surgery Guangdong Second Provincial General Hospital Guangzhou China

**Keywords:** telemedicine, health care delivery, prescription, cost, patient satisfaction, patient compliance

## Abstract

**Background:**

Health care maldistribution is a long-term problem in China. Telemedicine is an efficient way to deliver medical resources to remote areas; however, there are few studies on the effectiveness and challenges in providing health care from rural to urban areas in China.

**Objective:**

The objective was to describe the effectiveness and challenges of telemedicine for providing health care from Guangzhou to rural areas in Guangdong Province.

**Methods:**

We designed surveys and conducted them immediately after the consultation and 2-4 weeks later. Data were collected from June 2015 to May 2016 including patients’ demographic features, patient satisfaction, medicine effect, patient compliance, acceptability of prescription expenses, patients’ desire to revisit the department, the top 10 diseases, and self-reported difficulties in telemedicine experience. The monthly average prescription expense was described using a line chart. The monthly consultation and prescription, as well as monthly prescriptions of Western medicines and herbs, were described using a bar chart.

**Results:**

Women comprised majority (45,386/67,740, 67.00%) of participants and men comprised the minority (22,354/67,740, 33.00%). The top 3 diseases were upper respiratory diseases (12,371/36,311, 34.07%), laryngopharyngitis (4938/36,311, 13.60%), and menstrual disorders (4669/36,311, 12.86%). The monthly prescription for Western medicine was much more than that for Chinese herbs. The annual average medicine expense per prescription was 62.9 ¥. The participants’ perception of expense was acceptable (8775/12,450, 70.48%), mostly acceptable (2497/12,450, 20.01%), accepted but somewhat expensive (980/12,450, 7.9%), and unacceptable because of high cost (198/12,450, 1.6%). The surveys on patient satisfaction demonstrated very satisfied (55,687/67,740, 82.21%), satisfied (5891/67,740, 8.70%), basic satisfaction (3561/67,740, 5.26%), dissatisfaction (1454/67,740, 2.15%), and no comment (1147/67,740, 1.69%). Participants reported their treatment outcome as follows: full recovery (5008/12,450, 40.22%), recovering (4873/12,450, 39.14%), no effect (2153/12,450, 17.29%), or worsening (416/12,450, 3.3%). Approximately 89.01% (20,240/22,740) of participants will revisit the department, whereas 10.99% (2500/22,740) will not. Most patients complied with the doctors’ advice completely (5430/10,290, 52.77%), whereas the rest reported partial compliance (3684/10,290, 35.80%) or no compliance at all (1176/10,290, 11.43%). The participants reported poor computer skills (4980/22,740, 21.90%), transportation inconvenience (4670/22,740, 20.50%), unstable internet connection (3820/22,740, 16.80%), language barriers (3708/22,740, 16.30%), medication and medical hardware shortage (2459/22,740, 10.82%), tiring commute (2068/22,740, 9.08%), family care burdens (679/22,740, 3.0%), and other unclassified difficulties (356/22,740, 1.6%) as difficulties in using telemedicine.

**Conclusions:**

Telemedicine has a wide disease spectrum, similar to ordinary medicine in China. It saves costs, has high patient satisfaction and price acceptability, and can relieve disease and syndromes. However, certain problems need to be resolved. Telemedicine could be a feasible approach to address the health care maldistribution in rural China. This study may provide useful information for policy making and guidance for further telemedicine practice in China and other developing countries.

## Introduction

China is regarded as a traditionally agricultural society comprised of urban and rural areas with populations of 6.6 billion and 6.7 billion, respectively [1]. The maldistribution of health care resources between rural and urban areas has been an obvious problem to date despite the launch of medical reforms by the government [2-4]. It has been reported that the nationwide hospital bed ratio in the urban population was 0.087% compared with 0.018% in the rural population. The ratio of medical professionals in the urban population was 0.1% compared with 0.016% in the rural population [5], indicating that substantially more people live in rural areas but have fewer medical resources. Poor access to medical care was so common that peasants have had to spend a whole day registering and another several hours waiting to see a doctor. Some were even ignorant about their disease until they became seriously ill.

Under these circumstances, the Chinese government proposed the development of telemedicine service as an approach to optimize and redistribute medical resources in remote places [6]. The World Health Organization definition of telemedicine is the delivery of health care services, wherein distance is a critical factor, by all health care professionals using information and communication technologies for the exchange of valid information for the diagnosis, treatment, and prevention of diseases and injuries, research and evaluation, and for the continuing education of health care providers, all in the interest of advancing the health of individuals and their communities [7]. Previous research has confirmed the advantages of telemedicine, such as saving traveling time and reducing medical expenses [8,9], increasing patient satisfaction [9,10], effectiveness comparable to hospital face-to-face consultation [11], and improved management of chronic diseases such as diabetes and hypertension [8,12]. However, the effectiveness of telemedicine for the distribution of medical resources in rural China remains unclear. Guangdong Province is at the frontier of economic reform in China, and the Pearl River Delta region is more affluent than other rural areas.

In this study, we present the practice of telemedicine in a tertiary hospital in Guangdong Province that provides high-quality medical services to rural areas. We will describe the application, effectiveness, and difficulties of telemedicine practice for providing health care resources from Guangzhou to rural areas of Guangdong Province.

## Methods

### Study Design and Participants

**Figure 1:**
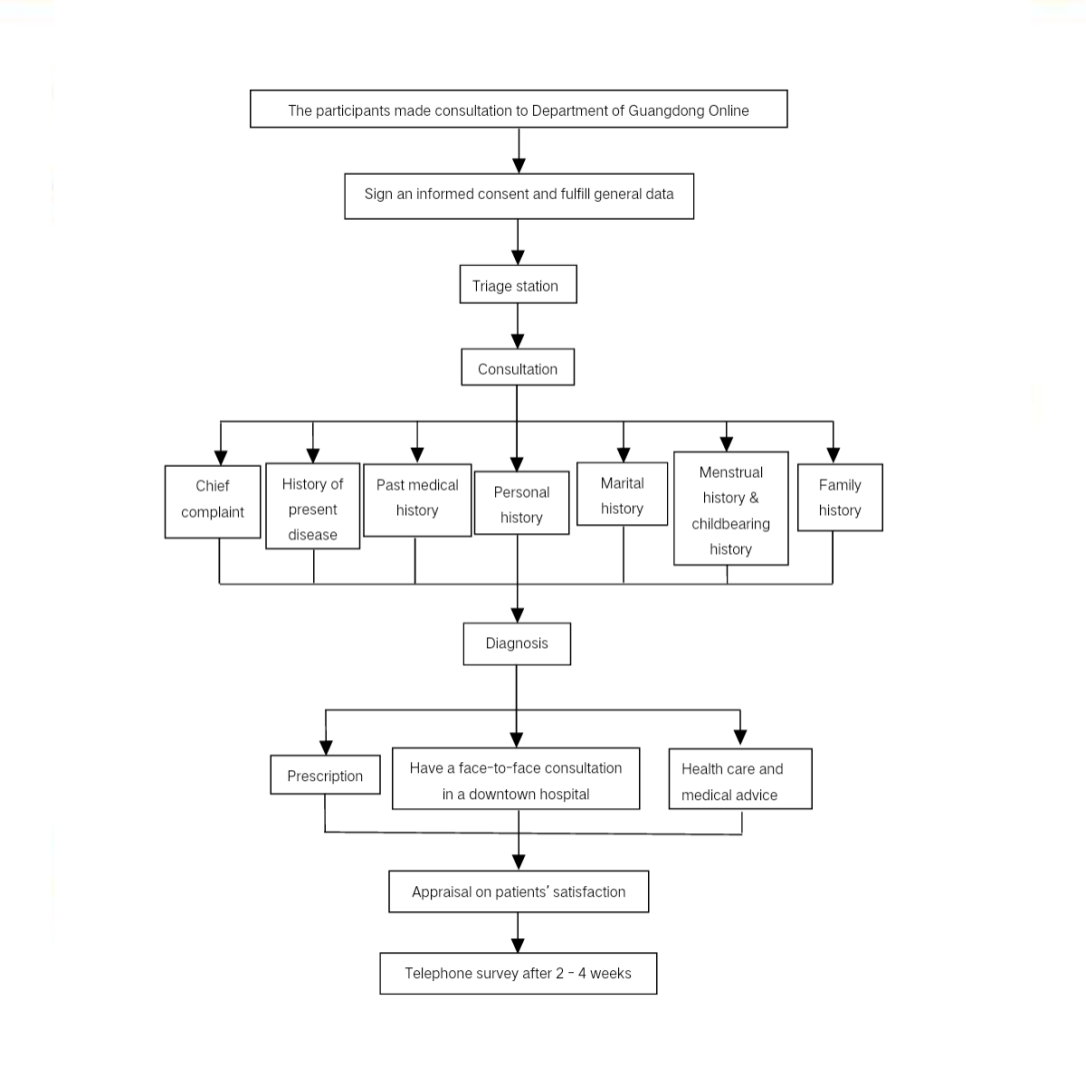
Flowchart for telemedicine consultation with Department of Guangdong Online Hopsital, Guangdong Second Provincial General Hospital.

The study enrolled participants who requested telemedicine consultation and medication at rural sanitary stations connected to the Department of Guangdong Online Hospital, Guangdong Second Provincial General Hospital, from June 2015 to May 2016. Each participant was informed of what telemedicine was and of the nature of the research and voluntarily signed a consent form before consultation. The participants consulted the remote medicine appliance system and registered with their demographic data including name, sex, age, telephone number, identity card number, historical medical record, and allergic history. The triage staff at the Department of Guangdong Online Hospital referred them to a relevant doctor after assessing and recording their vital signs at the rural sanitary stations. A diagnosis was provided by the doctor, and medical care was delivered to the sanitary station. The doctors prescribed medicine to the participants (medication prescription group), gave professional advice (medical advice group), or suggested that they have a face-to-face consultation with the doctors at a downtown hospital (face-to-face consultation group) depending on the participants’ diseases or syndromes. Prescribed medicines included Western medicines (Western medicine prescription group) and Chinese herbs (herb prescription group). For instance, a patient with a cough consulted with the Online Hospital at a sanitary station. The doctor diagnosed him with chronic bronchitis through inquiry, history medical record, and checking of his vital signs. The doctor prescribed medicine if he were confident of the diagnosis and considered the medicine necessary. The doctor gave professional suggestions such as the patient quit smoking and avoid allergens if he thought it was unnecessary for the patient to take medicine. However, if the doctor was unsure about the diagnosis or thought that the professional advice or medicine prescribed would not alleviate cough, he advised the patient to have a face-to-face consultation with a doctor at a downtown hospital. The communication was mainly conducted in Mandarin and Cantonese; some illiterate patients were assisted by staff at the rural sanitary stations. Satisfaction surveys were conducted immediately after the consultation, and telephone surveys on patient satisfaction, self-reported therapeutic effects, patients’ desire to revisit the Department of Guangdong Online Hospital, patients’ compliance with doctors’ advice, and self-reported difficulties with their telemedicine experience were conducted 2 to 4 weeks after the consultation (Figure 1). Two staff members from the department underwent advanced training to conduct telephone surveys. A full ethics review of the research protocol was conducted by the Institutional Review Board at the Guangdong Second Provincial General Hospital.

**Figure 2:**
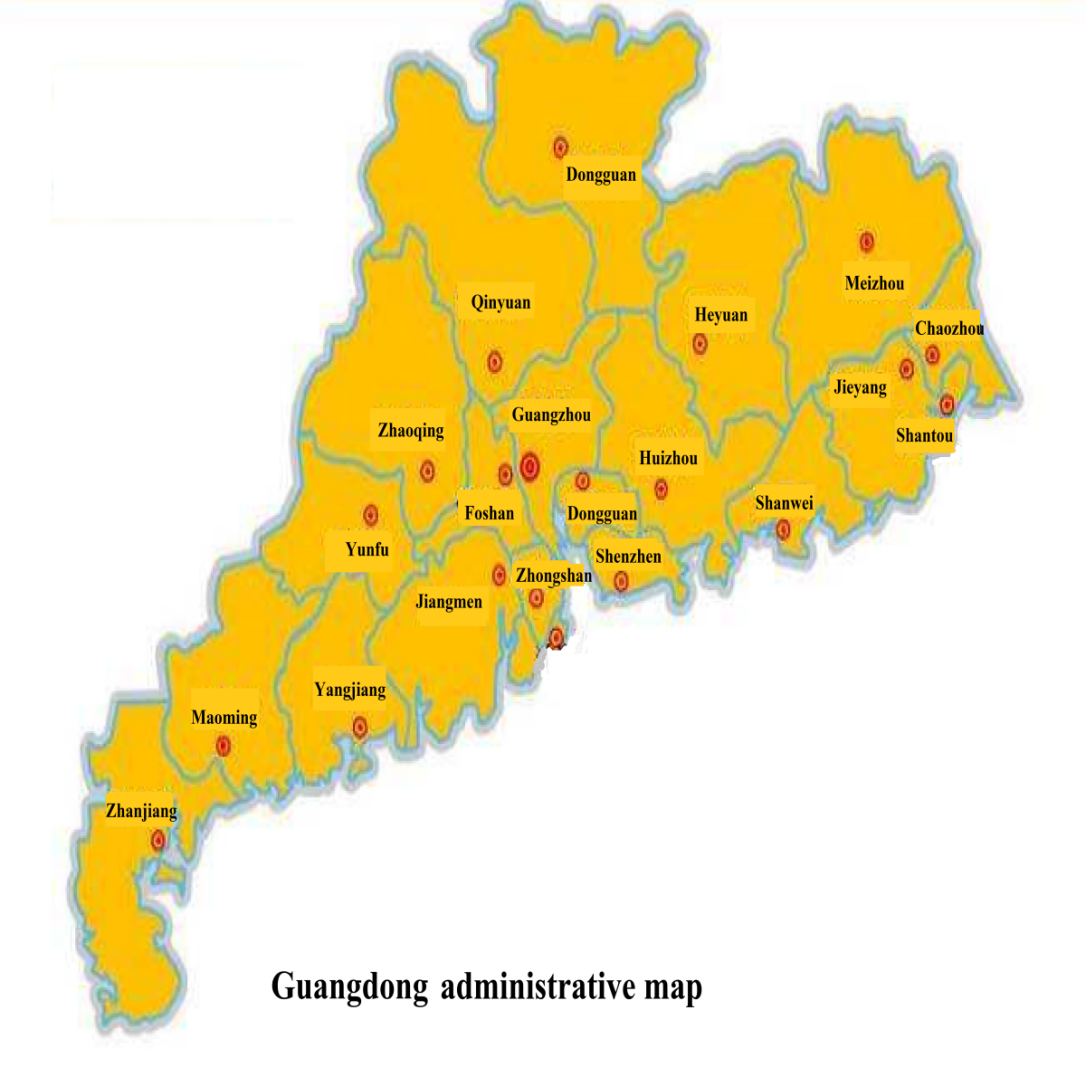
Guangdong administrative map.

This study was launched by the Department of Guangdong Online Hospital, Guangdong Second Provincial General Hospital [13] the first telemedicine institution authorized by the Chinese government. The Department of Guangdong Online Hospital consisting of 24 clinics worked simultaneously with doctors from internal medicine, pediatrics, traditional Chinese medicine, dermatology, rehabilitation, and gynecology departments at the hospital. A medical technology company supported the Web-based consultation platform services including visual telephone, Web-based prescription services, electronic anatomic diagrams, triage, blood pressure and blood glucose measurement, Web-based auscultation, and geographic maps of the patients’ locations. The telemedicine network covered approximately 300 rural sanitary stations in the rural areas of Maoming, Jieyang, Zhaoqing, Huizhou, Shantou, Zhanjiang, Zhongshan, Jiangmen, Meizhou, Yunfu, Heyuan, Yangjiang, Shaoguan, Chaozhou, and Shanwei (Figure 2 and Table 1).

**Table 1:** Distance to Guangzhou and population of rural regions.

Rural regions	Distance to Guangzhou (km)	Population, n
Mao ming	340.5	5,817,753
Jie yang	430.4	5,877,025
Zhao qing	97.1	3,918,085
Hui zhou	144.2	4,597,002
Shan tou	439.3	5,391,028
Zhan jiang	416.4	6,993,304
Zhong shan	83.4	3,120,884
Jiang men	91.3	4,448,871
Mei zhou	390	4,240,139
Yun fu	143.8	2,360,128
He yuan	199.6	2,953,019
Yang jiang	223.6	2,421,812
Shao guan	222.4	2,826,612
Chao zhou	498	2,669,844
Shan wei	274.2	2,935,717

**Participants’ (medication prescription group) attitudes toward telemedicine.**

Name: Sex: Age:

Identity number: Telephone number:

Residence place: Public clinic:

Please check the option that fits for you.

1. What kind of medicine did you obtain from the doctors?

Western medicine ( ); Chinese herbs ( )

2. What was the curative effect of the drug prescribed by the doctor?

Cured ( ); got better ( ); no use ( ); got worse ( )

3. What is your acceptance of the medical expense?

Completely acceptable ( ); mostly acceptable ( ); acceptable but somewhat expensive ( ); unacceptable due to high cost ( )

4. Do you think telemedicine is useful?

Very useful ( ); somewhat useful ( ); not very useful ( ); no use ( )

5. Will you visit the online hospital again?

Yes ( ); no ( )

6. What difficulties you have had in your telemedicine experience?

### Survey Instruments

The survey instruments used in this study consisted of 3 evaluation charts. The charts were formulated according to the following 3 categories of service received by participants: medication prescriptions (Textbox 1), medical advice (Textbox 2), and referral to a doctor at a downtown hospital (Textbox 3). Each participant was interviewed and evaluated voluntarily according to the medical service received.

The questionnaires contained the basic information of the participants. Each chart contained 5 to 6 questions focusing on opinions and attitudes about telemedicine, including effectiveness of treatment, willingness to revisit, acceptance of medical expenses, and difficulties with the telemedicine experience.

**Participants’ (medical advice group) attitudes toward telemedicine.**

Name: Sex: Age:

Identity number: Telephone number:

Residence place: Public clinic:

Please check the option that fits for you.

1. Did you follow the doctor’s advice completely?

Completely ( ); partially ( ); just listened to the advice ( )

2. What are your opinions on the usefulness of the doctor’s advice?

Very useful ( ); useful ( ); no use ( ); got worse ( )

3. Do you think that telemedicine is useful?

Very useful ( ); useful ( ); not too useful ( ); no use ( )

4. Will you visit online hospital again?

Yes ( ); no ( )

5. What difficulties have you had in your telemedicine experience?

**Participants’ (face-to-face consultation group) compliance with Web-based physician advice.**

Name: Sex: Age:

Identity number: Telephone number:

Residence place: Public clinic:

Please tick the option that fit for you.

1. Did you follow the doctor’s advice to have a face-to-face consultation in a downtown hospital?

Yes ( ); no ( )

2. What was the doctors’ diagnosis?

3. What therapy did you receive at the downtown hospital?

Medicine ( ); surgery ( ); rehabilitation ( ); psychiatry ( ); others ( )

4. What was the outcome of the telemedicine?

Cured ( ); got better ( ); no curative effect ( ); got worse ( )

5. Do you think that telemedicine is useful?

Very useful ( ); useful ( ); no use ( ); got worse ( )

6. Would you visit the Department of Guangdong Online Hospital again?

Yes ( ); no ( )

### Statistical Analysis

Five quantitative and qualitative variables were categorized and calculated according to data collected from the 15 rural regions shown in Figure 1. The demographic characteristic and top 10 diseases and syndrome were collected from annual surveys, whereas patient satisfaction, prescription amount (the number of times patients received prescription orders), consultation amount (the number of times they received consultations without prescriptions), and the average prescription expense were analyzed monthly. The questionnaire items about medication effectiveness, acceptance of prescription expense, attitudes toward remote medicine, and difficulties with the telemedicine experience were also investigated. The surveys were conducted by telephone. Data including patients’ demographic features, patient satisfaction, medicine effect, patients’ compliance with doctors’ advices, patients’ acceptance of prescription expense, patients’ desire to revisit the Department of Guangdong Online Hospital, the top 10 diseases and self-reported difficulties in telemedicine experience that were described in terms of frequency and percentages (n, %). The monthly average prescription expense was described using a line chart. Monthly consultation and prescription amount, as well as monthly prescriptions of Western medicine and herbs, were described using a bar chart. The age distribution of the consultation population was described using a pie chart. Statistical analysis was performed with SPSS Version 22.0 statistic software package.

## Results

### Demographic Characteristics of Telemedicine Consultation

From June 2015 to May 2016, approximately 67,740 participants had consultations at the Department of Guangdong Online Hospital, Guangdong Second Provincial General Hospital. Approximately 22,740 of these participants were interviewed voluntarily. The number of participants in the medical prescription group was much greater than that in the medical and face-to-face consultation groups. Women comprised majority (45,386/67,740, 67.00%) of the participants and men comprised the minority (22,354/67,740, 33.00%, Table 2). Participants between 20 and 30 years old were more inclined to use telemedicine (24,269/67,740, 35.83%) compared with the others (Table 3).

### Diseases, Consultations, Prescriptions, and Medicine Expenses

#### Disease Spectrum

The top 10 diseases included respiratory ailments, gynecological conditions, sleep disorders, and pain syndrome. The top 3 diseases and syndromes of telemedicine consultation in rural areas were upper respiratory ailments (12,371/36,311, 34.07%), laryngopharyngitis (4938/36,311, 13.60%), and menstrual disorder (4669/36,311, 12.86%), as seen in Table 4.

**Table 2:** Demographic characteristics of telemedicine users (N=67,740).

Demographic characteristics	Medical prescription group (n=35,021)	Medical advice group (n=21,887)	Face-to-face consultation group (n=10,832)	Total
Male, n (%)	10,059 (28.72)	8271 (37.79)	4024 (37.15)	22,354 (33.00)
Female, n (%)	24,962 (71.28)	13,616 (62.21)	6808 (62.85)	45,386 (67.00)
Age, mean (SD)	34.05 (15.45)	35.75 (20.40)	36.81 (14.72)	34.76 (17.06)

**Table 3:** Age distribution of consultation population (N=67,740).

Age (years)	n (%)
<10	2618 (3.86)
11-20	3657 (5.40)
21-30	24,269 (35.83)
31-40	14,060 (20.76)
41-50	10,808 (15.95)
51-60	7816 (11.54)
61-70	3069 (4.53)
>70	1443 (2.13)

**Table 4:** Top 10 diseases and syndromes in telemedicine consultations in rural regions (N=36,311).

Ranking	Disease or syndromes	n (%)
1	Upper respiratory infection	12,371 (34.07)
2	Laryngopharyngitis	4938 (13.60)
3	menstrual disorder	4669 (12.86)
4	Cough	3728 (10.27)
5	sleep disorder	3132 (8.63)
6	Gastritis	1969 (5.42)
7	Dyspepsia	1812 (4.99)
8	Colpitis	1400 (3.86)
9	Dysmenorrheal	1258 (3.45)
10	Osphyalgia	1034 (2.85)

**Figure 3:**
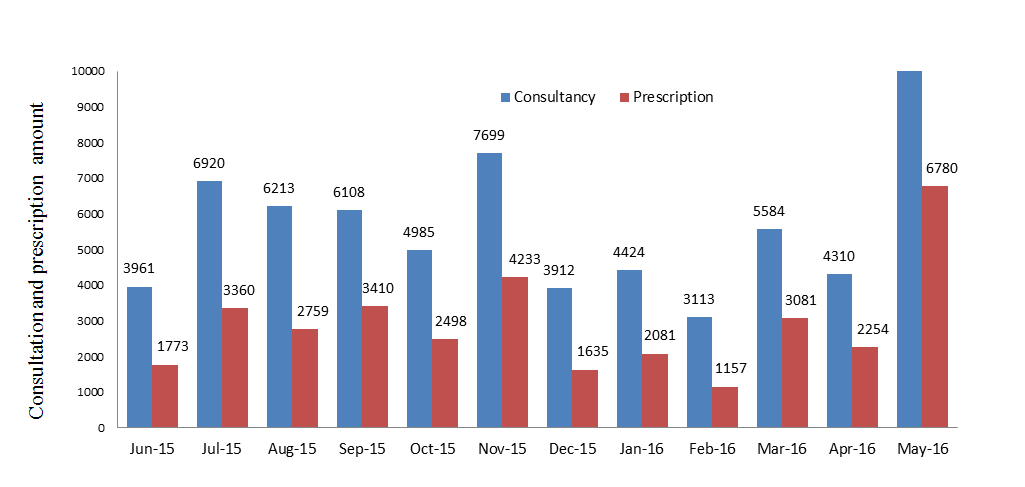
Monthly consultations and prescriptions.

#### Consultations and Prescriptions

**Figure 4:**
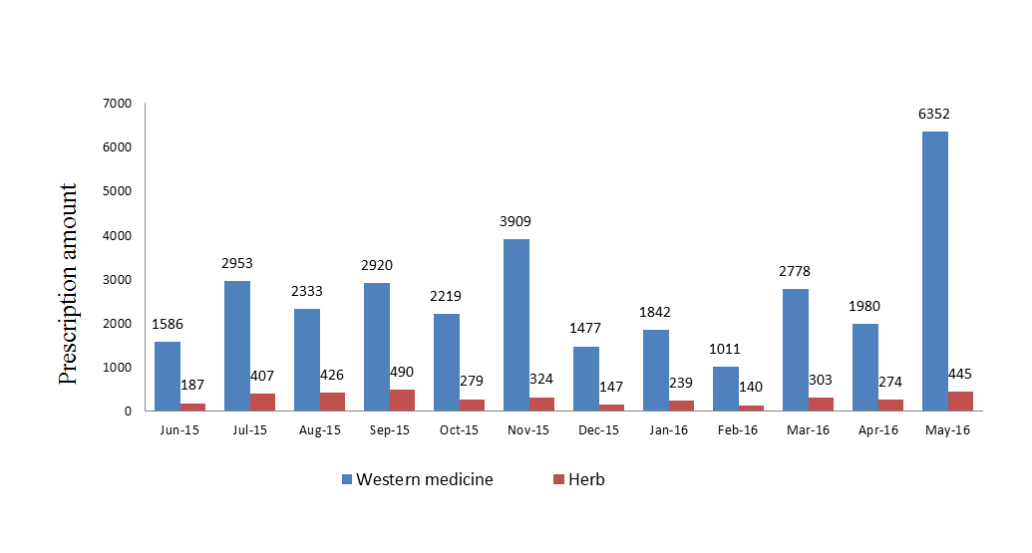
Monthly prescriptions for Western medicine and herbs.

The prescription amount (35,021) was lower than the total consultation amount (67,740) from June 2015 to May 2016, and the prescription amount was lower than the consultation amount every month (Figure 3). The total number of prescriptions for Western medicines (31,360) was much more than the number of prescriptions for Chinese herbs (3661) from June 2015 to May 2016, and the number of monthly prescriptions for Western medicines was much greater than that for Chinese herbs (Figure 4).

#### Monthly Average Medicine Expense Per Prescription

**Figure 5:**
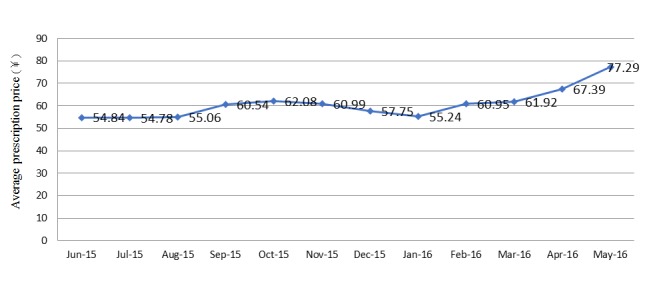
Monthly average prescription expenditures.

The monthly average medicine expense per prescription was lowest in June 2015 (54.84 ¥) and highest in May 2016 (77.29 ¥) but did not change substantially within a year (Figure 5). The annual average medicine expense per prescription was 62.9 ¥.

### Results of Telephone Surveys

#### Patient Satisfaction

The surveys on patient satisfaction demonstrated that most patients were very satisfied (55,687/67,740, 82.20%), whereas few were satisfied (5891/67,740, 8.70%), basically satisfied (3561/67,740, 5.26%), dissatisfied (1454/67,740, 2.15%), or had no comment (1147/67,740, 1.69%). The percentages of those who were very satisfied, satisfied, and basic satisfied in the face-to-face consultation group (10,527/10,832, 97.10%) was higher than those in the medical prescription group (33,622/35,021, 96.00%) and the medical advice group (20,990/21,887, 95.90%), as seen in Table 5.

#### Medical Expense Acceptance

Most participants perceived the telemedicine expense to be acceptable (8775/12,450, 70.50%) or mostly acceptable (2497/12,450, 20.01%) with fewer perceiving it to be acceptable but somewhat expensive (980/12,450, 7.9%) or unacceptably expensive (198/12,450, 1.6%). The percentages of those who found their expenses completely acceptable, mostly acceptable, or acceptable but somewhat expensive in the Western medicine prescription group (11,103/11,271, 98.5%) were higher than in the herbal prescription group (1149/1179, 97.46%), as seen in Table 6.

#### Self-Reported Therapeutic Effects

A majority of participants reported positive treatment outcomes (full recovery: 5008/12,450, 40.22% and recovering: 4873/12,450, 39.14%); fewer reported no effect (2153/12,450, 17.29%) or worsening conditions (416/12,450, 3.3%). The percentages of full recovery and recovery in the Western medicine prescription group (8952/11,271, 79.43%) were the same as those in the herbal prescription group (929/1179, 78.80%), as seen in Table 7.

#### Patient Compliance

Patients’ compliance with doctors’ advice included complete compliance (5430/10,290, 52.77%), partial compliance (3684/10,290, 35.80%) with doctor advice and no compliance at all (1176/10,290, 11.43%). Approximately half reported complete compliance in both the medical advice group and the face-to-face consultation group. Nearly half complied with the doctors’ advice partially and just listened to the advice without action in the medical advice group (3235/6907, 46.84%) and the face-to-face consultation group (1625/3383, 48.03%), as seen in Table 8.

#### Patients’ Desire to Revisit the Online Hospital

Approximately 89.00% (20,240/22,740) of the participants were willing to revisit the Online Hospital, whereas 10.99% (2500/22,740) would not. Most of the participants in the medical prescription group (11,078/12,450, 88.98%) would revisit it, as would those in the medical advice group (6182/6907, 89.50%) and face-to-face consultation group (2982/3383, 88.10%), however, the rest would not revisit it, as seen in Table 9.

**Table 5:** Patient satisfaction rates in the 3 groups (N=67,740).

Patient satisfaction	Medical prescription group (n=35,021), n (%)	Medical advice group (n=21,887), n (%)	Face-to-face consultation group (n=10,832), n (%)	Total, n (%)
Very satisfied	28,718 (82.00)	17,838 (81.50)	9131 (84.30)	55,687 (82.21)
Satisfied	3152 (9.00)	1860 (8.50)	879 (8.11)	5891 (8.70)
Basic satisfied	1752 (5.00)	1292 (5.90)	517 (4.77)	3561 (5.26)
Unsatisfied	701 (2.0)	547 (2.5)	206 (1.90)	1454 (2.14)
No comment	698 (2.0)	350 (1.6)	99 (0.91)	1147 (1.69)

**Table 6:** Annual expense acceptance (N=12,450).

Expense acceptance	Western medicine prescription group (n=11,271), n (%)	Herb prescription group (n=1179), n (%)	Total, n (%)
Completely acceptable	7956 (70.59)	819 (69.47)	8775 (70.48)
Mostly acceptable	2262 (20.07)	235 (19.93)	2497 (20.02)
Acceptable but somewhat expensive	885 (7.85)	95 (8.06)	980 (7.9)
Unacceptable due to the high cost	168 (1.49)	30 (2.54)	198 (1.6)

**Table 7:** Self-reported therapeutic effects (N=12,450).

Treatment outcome	Western medicine prescription group (n=11,271), n (%)	Herb prescription group (n=1179), n (%)	Total, n (%)
Full recovery	4528 (40.17)	480 (40.71)	5008 (40.22)
Recovery	4424 (39.25)	449 (38.08)	4873 (39.19)
No effect	1933 (17.15)	220 (18.66)	2153 (17.29)
Worsening	386 (3.42)	30 (2.54)	416 (3.3)

**Table 8:** Patient compliance with doctors’ advice (N=10,290).

Patient compliance	Medical advice group (n=6907), n (%)	Face-to-face consultation group (n=3383), n (%)	Total, n (%)
Completely	3672 (53.16)	1758 (51.97)	5430 (52.77)
Partially	2442 (35.36)	1242 (36.71)	3684 (35.80)
Just listen to the advice	793 (11.48)	383 (11.32)	1176 (11.43)

**Table 9:** Patients’ desire to revisit the Department of Guangdong Online Hospital (N=22,740).

Whether to revisit	Medical prescription group (n=12,450), n (%)	Medical advice group (n=6905), n (%)	Face-to-face consultation group (n=3385), n (%)	Total, n (%)
Yes, I will revisit the online hospital	11,078 (88.98)	6180 (89.50)	2982 (88.)	20,240 (89.01)
No, I will not revisit the online hospital	1372 (11.02)	725 (10.50)	403 (11.90)	2500 (10.99)

**Table 10:** Self-reported difficulties with the telemedicine experience (N=22,740).

Difficulties in telemedicine experience	n (%)
Language barrier	3708 (16.30)
Tiring commute	2068 (9.08)
Transportation inconvenience	4670 (20.50)
Medication shortages	1282 (5.64)
Unstable internet connection	3820 (16.80)
Poor computer skills	4980 (21.90)
Family care burden	679 (3.0)
Medical hardware shortage	1177 (5.18)
Others unclassified difficulties	356 (1.6)

#### Self-Reported Difficulties With Telemedicine Experience

The top 3 self-reported difficulties by the participants included poor computer skills (4980/22,740, 21.90%), transportation inconvenience (4670/22,740, 20.50%), and an unstable internet connection (3820/22,740, 16.80%), whereas they also complained about medical hardware and medicine shortages, language barriers, and tiring commute (Table 10).

## Discussion

### Principal Findings

Data obtained in previous studies indicated that telemedicine could significantly improve patient satisfaction, save costs and time compared with conventional means, and increase access to health care resources for rural patients [9,14]. The results of our study were almost consistent with the findings of previous research but presented more problem-oriented and detailed results. In this study, we found that telemedicine has a wide disease spectrum, similar to ordinary medicine in China. It saves costs, has high patient satisfaction and price acceptability, and can relieve disease and syndromes. However, challenges such as poor computer skills, transportation inconvenience, language barriers, unstable internet connection, and medication and hardware shortage at sanitary stations need to be resolved.

### Demographic Characteristics of Telemedicine Consultation

We noted some demographic characteristics concerning the inclination to use telemedicine. Participants between 20 and 40 years old accounted for more than half of the consultation, and women were more inclined than men to use the internet to obtain medical services, which was not reported previously. These findings indicated that people in this age spectrum have more open attitudes toward new ways of receiving health care. They have better computer skills and language abilities than seniors and the young, which might be the reasons for the inclination. The rural migrant workers account for most of the migrant population in China. The percentages of rural female and male migrant workers were 47% and 53%, respectively [15]. This means that more female farmers stay at home compared with men. Women were the main users of telemedicine because their spouses were working in the cities which is a characteristic pattern in China.

### Telemedicine Practice in Rural Areas

#### Disease Spectrum

Our research found that the top 3 diseases and syndromes treated by telemedicine were mainly upper respiratory diseases, Laryngopharyngitis and menstrual disorder, a pattern comparable with that reported in the health medical yearbook of China in 2009 [16]. This finding could guide disease prevention strategies in the rural regions of Guangdong Province.

#### Consultation and Prescription

Monthly consultation and prescription showed an abnormal distribution. The consultation services included prescriptions and advices. Some of the participants obtained prescriptions, whereas the others received professional advice from doctors. The number of prescriptions was lower than the number of visits every month. Both Western medicine and herbs were prescribed with the former prescribed much more than the latter, expanding the range of telemedicine treatment reported previously [17-19]. Traditional Chinese medicine (TCM) has had deep and wide grassroots following in rural China [20] and still plays an important role there. Statistics indicate that the number of consultations at rural sanitary stations for TCM service was 51,707,000 in 2012 [21]. Chinese herbs were undoubtedly popular in telemedicine practice in rural areas. The TCM services were delivered from a tertiary hospital to sanitary stations via telemedicine. This study, therefore, provided a good example of a combined treatment using both Western medicine and herbs for dealing with health problems by telemedicine in rural regions.

#### Prescription Expenses

We calculated that the annual average prescription price was 62.9 ¥, which was much lower than the average outpatient prescription expenditure of 291.3 ¥ in tertiary public hospitals and 189.5 ¥ in secondary public hospitals according to the latest data from January 2016 to October 2016 from the website of the National Health and Family Planning Commission of the People’s Republic of China [22]. Therefore, the results indicated that telemedicine could save costs compared with conventional medicine and serve as a feasible solution to solve the problem of health care costs.

### Effectiveness and Challenges

#### Effectiveness

Telemedicine had a high patient satisfaction rate. A survey of patient satisfaction was conducted immediately after the consultations. Approximately vast majority (55,687/67,740, 82.21%) of the participants were very satisfied with their telemedicine experience, whereas minority (1454/67,740, 2.14%) was not satisfied. The results of this research were comparable with those of a previous study that found a good patient satisfaction rate with telemedicine in rural regions [10]. A survey on patient satisfaction with conventional medicine among peasants by the statistics center of the National Health and Family Planning Commission demonstrated that a high proportion of peasants were satisfied (80%), whereas a small fraction was unsatisfied (1.5%), which was lower than the patient satisfaction rate in telemedicine practice [23]. This study indicated that the telemedicine prescription expense was highly acceptable by rural patients with a rate of acceptance (11,272/12,450, 90.50%) that was higher than that for conventional medicine in rural areas (40%) [23]. The consultation was free, and patients only had to pay for medication, which was different from face-to-face consultations in hospitals in China. Our survey results implied that a large proportion of participants believed that telemedicine could save costs. Because the annual average prescription expense was much lower than the outpatient prescription expense assessed by the National Bureau of Statistics of China [22], we determined that telemedicine could save costs, which was consistent with Thaker’s findings [24], but diverged from those of Upatising [25]. Our study indicated that telemedicine could help alleviate or even cure diseases or syndromes, thus improving treatment outcomes, which were consistent with the findings of Tso [26] and Muller [27]. We also found that approximately 89% of the participants would like to revisit the Department of Guangdong Online Hospital. The number of participants who reported complete adherence to physician’s advice was almost equal to that of those reporting partial compliance and no compliance, which was consistent with Bateman’s research [28]. The findings of our study indicated that telemedicine was popular but did not have a good compliance. Therefore, more research and efforts are needed to improve patient compliance.

#### Challenges

We also assessed the difficulties associated with the telemedicine experience. Opinions from 22,740 participants on the problems they encountered and included poor patient computer skills, transportation problems, an unstable internet connection, and language communication barriers. The percentage of the peasants who were illiterate and had less than a middle school education was 91.7% [15]; therefore, acquisition of computer skills and Mandarin or Cantonese would undoubtedly be hardship for them, especially for the seniors. The older participants tended to consider poor computer skills and poor oral expression and understanding of Mandarin, which were quite different from their local dialects, as the biggest barriers in their telemedicine experiences; as a result, in most cases, they needed assistance from the sanitary station. The participants also complained of the inconvenience of traveling several miles without public transportation to the downtown hospitals. Some complained of an unstable internet connection with intermittent visual and auditory signals hindering smooth communication with doctors. Some complained of exhaustive journeys from their home to sanitary station without public transportation. The participants, especially women, mentioned that no one could take care of their children, parents, farmland, and livestock if they traveled downtown for telemedicine services. Some complained that the sanitary stations lacked the medicines prescribed by the doctors, requiring them to seek substitute medications or return home empty handed. Some also complained that the pharmacies lacked the usual medical devices, such as a hematomanometer and glucose meter. This research was the first study to our knowledge to investigate the difficulties with telemedicine experience for people in rural regions in China; it could thus provide useful guidance for future telemedicine practice in rural areas.

### Background on Telemedicine Practice in Rural China

The maldistribution of medicine and hygiene resources between rural and urban regions has been evident despite several stages of medical reforms initiated in 1985. The rural population accounted for a large portion of the population but had fewer medical resources of both medical institutions and staffs than people in cities. The statistics reveal that the total number of people diagnosed and treated in medical and hygiene institutions in China was 64.2 billion, an increase of 2%, and the number of people in hospitals was 26.3 billion, an increase of 5%, but in grass-root medical institutes, the number was 35.6 billion, a decrease of 1% compared with the same period in 2016 [29]. This implies that a large number of rural people would seek health care from urban hospitals but not in their places of residential areas. As a result, the Chinese government launched official documents requiring and advocating telemedicine as an important approach for solving medical resource inequality between urban and rural regions and facilitate the ability of rural people to obtain medical services in their areas through local medicine and hygiene administration departments to resolve difficulties in access to better and sufficient medical services for rural residents [6]. The Department of Guangdong Online Hospital, Guangdong Second Provincial General Hospital, the first remote medical institution authorized by the National Health and Family Planning Commission, has endeavored to deliver health care to rural regions through telemedicine practice on which our research was based, indicating that adding telemedicine to rural sanitary stations was a feasible way to solve difficulties in access to high-quality medical services in tertiary hospitals for rural residents.

### Limitations

The results of this study could guide further promotion and advocacy of telemedicine for delivering better medical services from larger cities to remote and rural regions in China and other developing countries. However, some limitations are worth noting. Some of the participants could not be contacted for follow-up telephone interviews because of wrong telephone numbers or their refusal to answer questions.

### Conclusions and Future Directions

Telemedicine has a wide disease spectrum similar to that addressed by ordinary medicine in China. It could save costs, provide high patient satisfaction, popularity and price acceptance, and help cure or relieve diseases and syndromes. However, problems remain that must be resolved, including poor computer skills, transportation inconvenience, language communication barriers, unstable internet connection, and medicine and hardware shortage at sanitary stations. Telemedicine could serve as a feasible approach to addressing the maldistribution of health care resources in rural China. The results could guide further promotion and advocacy of telemedicine practice in rural regions of China and other developing countries. Future research should focus on providing a smooth and stable telemedicine network, resolving transportation inconvenience, improving patient compliance and helping the elderly obtain telemedicine services in rural regions.

## Abbreviations

TCM: traditional Chinese medicine
